# PROTOCOL: The effectiveness of interventions for reducing violence against children: An evidence and gap map in low‐ and middle‐income countries

**DOI:** 10.1002/cl2.1040

**Published:** 2019-09-05

**Authors:** Prachi Pundir, Ashrita Saran, Howard White, Jill Adona, Ramya Subrahmanian

**Affiliations:** ^1^ Campbell Collaboration 2nd Floor, West Wing, ISID Complex, Vasant Kunj Delhi 110070 India; ^2^ Asian Development Bank Manila Philippines Philippines; ^3^ UNICEF Office of Research‐ Innocenti Piazza SS. Annunziata Florence 12 50122 Italy

## 1 BACKGROUND

### 1.1 The problem, condition or issue

Violence against children includes all forms of violence under 18 years old, whether perpetrated by parents or other caregivers, peers, romantic partners, or strangers (World Health Organization [WHO, 2018]). As defined by The United Nations Children's Fund (UNICEF), violence is, “all forms of physical or mental violence, injury or abuse, neglect or negligent treatment, maltreatment or exploitation, including sexual abuse” (UNICEF, 2017). It includes maltreatment, bullying, youth violence, intimate partner violence, sexual violence and emotional or psychological violence (detailed definition given in Appendix 1).

More than one billion children—half the children in the world—are victims of violence every year (Hillis et al., 2016). Global Burden of Disease estimates that 91.4% of deaths due to interpersonal violence occur in low‐ and middle‐income countries (LMICs) and the rate of collective violence being 10 times higher in LMICs than high‐income countries (HICs) (Mercy et al., 2017).

Violence, exploitation and abuse against children occur in the homes, families, schools, care and justice systems, workplaces and communities across all contexts, including as a result of conflict and natural disasters (UNICEF, 2011). Children are especially vulnerable to abuse, exploitation and trafficking (collective violence) during emergencies and armed conflicts. Children in different setting are exposed to various forms of violence including sexual abuse, armed violence, trafficking, child labour, gender‐based violence, gang violence, peer‐violence, corporal punishments, cyber bullying, female genital mutilation, violence in war/conflict affected region and child marriage (UNICEF, 2010).

A few striking features of childhood violence have drawn substantial international attention in recent years. First, violence exposure starts early in childhood through experience of corporal punishment as early as age. Second, much of the violence experienced by children is at the hands of adults who are typically to be found within a circle of trust and caregiving—parents, teachers, neighbours and authority figures. Third, there is also a striking rise in peer violence as children grow older and violence spills into peer relationships including through bullying [offline and online], dating and intimate partner violence, as well as gang violence (Know Violence in Childhood, 2017).

Evidence suggests that certain risk factors for violence may manifest differently across gender and age groups. WHO estimates that the highest child homicide rates occur in adolescents, especially boys, aged 15–17 years and among children 0–4 years old (WHO, 2004). A 2011 review estimated the global lifetime prevalence of childhood sexual abuse to be about 18% for girls compared with 8% for boys (Stoltenborgh, Van Ijzendoorn, Euser, and Bakermans‐Kranenburg, 2011). Girls all over the world are victims of child marriage, forced pregnancy, female genital mutilation, trafficking and child labour. The situation is alarming in LMICs because of higher rates of violence against girls as compared to HICs (WHO, 2013). In schools and other education settings boys seem to be more at risk than girls for harsh physical punishment and abuse. As per Global School‐based Student Health Survey (GSHS) reports of physical violence among students aged 13–15 are relatively common and are more in boys than girls (GSHS, 2013). Children in war‐affected regions of the world are vulnerable and easy targets for coercion. In 2016, the United Nations (UN) verified over 20,000 incidents of child violation in regions of armed‐conflict (UN, 2017).

**Figure 1:**
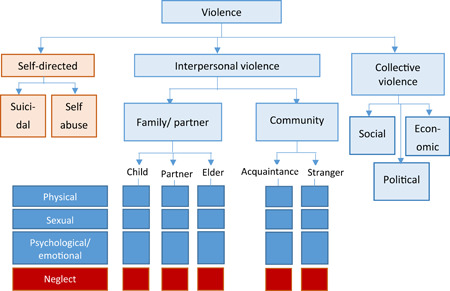
Typology of violence (Source: WHO, 2002), and areas (in blue) covered by the present Evidence and Gap map (EGM) [Color figure can be viewed at wileyonlinelibrary.com]

Figure 1 describes the classification of violence as stated in World report on Violence and Health (WHO, 2002). According to the report, violence is categorised into self‐directed violence, interpersonal violence and collective violence based on victim‐perpetrator relationship. Interpersonal violence includes family, intimate partner and community violence whereas collective violence encompasses larger group of people such as social, political and economic violence (Krug, Dahlberg, Mercy, Zwi & Lozano, 2002). The focus of present Evidence and Gap map (EGM) is only on interpersonal and collective violence against children. This map excludes self‐directed violence and neglect as this is the scope of an on‐going EGM on child abuse and neglect in LMICs (Sinha, Radhika, Jha & John, 2018). Structural violence is also beyond the scope of this map as the factors leading to and interventions to reduce/prevent such form of violence are far more complex than explanations would imply.

Violence experienced by children is of concern because of the serious intergenerational impacts on the future wellbeing of children. Experience of violence during childhood increases the risk of becoming victims or perpetrators of violence during adulthood (WHO, 2007). Violence can negatively affect physical, mental, sexual, and reproductive health, and may increase the risk of acquiring human immunodeficiency virus (HIV) in some settings (WHO, 2017). Violence against children is associated with poor education outcomes, economic insecurity including food insecurity, parental unemployment, inadequate housing and other basic necessities for children and families in LMICs (Peterman, Neijhoft, Cook, & Palmero, 2017). The global costs related to physical, psychological and sexual violence have been estimated to be between 3% and 8% of global GDP (Pereznieto, Montes, Langston, & Routier, 2014).

Although experiencing violence in childhood impacts lifelong health and well‐being, it is often preventable (World Health Organization, 2018). As part of the post‐2015 sustainable development goals (SDG) agenda, the UN issued a global call‐to‐action: to eliminate violence against children (United Nations Development Programme [UNDP, 2017]). In recognition of this, a specific target [SDG 16.2] was included in the 2030 Agenda for Sustainable Development giving renewed impetus towards the realisation of the right of every child to live free from fear, neglect, abuse and exploitation. Several other SDG targets address specific forms of violence such as eliminating violence against women and girls in public and private spheres [SDG 5.2], eliminating harm towards children such as child marriage and female genital mutilation [target 5.3], promotion of nonviolent educational environment [SDG 4.7] and the eradication of child labour, including the recruitment and use of child soldiers [target 8.7]. The year 2019 also marks the 30th anniversary of the adoption of the UN Convention on the Rights of the Child (CRC), which provides an important opportunity to gain a better understanding of the nature, extent and causes of violence against children.

Finally, the launch of the Global Partnership to End Violence against Children in 2016, serves as a global platform whose aim is “ending violence against children in every country, every community and every family” (End Violence Against Children, 2018). As part of the SDGs, the UN General Assembly has made a global commitment to ending all forms of violence against children. A technical package supporting seven evidence‐based strategies to end violence against children—INSPIRE—developed by the WHO and nine other international agencies and initiatives, has been widely promoted and adopted as an essential tool in supporting national investments and actions towards realising this commitment (WHO‐INSPIRE, 2016).

### 1.2 Why it is important to develop the EGM

In response to above there is an increased need to invest in generating sound evidence on effective strategies to prevent or end violence against children, in order to strengthen the evidence to policy architecture in this area. There has been a substantial increase in recent years in pilot programmes testing violence prevention strategies, many of which have contributed to the strategies prioritised in the INSPIRE technical package. However, there are still many gaps in the evidence base from these programmes, including geographical gaps, thematic gaps and missing information that constrain the building of a concerted evidence‐informed policy and investment agenda. EGMs can contribute by providing an overview of available quality studies, identifying gaps and thereby supporting the prioritisation of global evidence synthesis needs and primary data collection.

In order to move towards the ambitious targets laid out in the SDGs, it is likely that substantial improvements in resource allocation will be needed to promote interventions which are effective in improving outcomes in particular contexts. The purpose of this evidence and gap map is to assist policy‐makers and practitioners in gaining access to evidence on the effectiveness intervention to reduce violence against children. This will also help to identify research gaps with little or no evidence synthesis and to provide a resource for informing policy and practice. The EGM does not replace other primary research or address specific methodological or other gaps, but lays out an assessment of evidence availability in order to guide further research investment.

### 1.3 Conceptual framework of the EGM

Violence prevention is a complex field, involving a variety of strategies addressing different risk factors that are enmeshed in familial and social relationships, determined often by power inequalities as well as learned social behaviours and norms. In order to build on advances in the field of violence prevention, this EGM draws on the same framework developed by WHO and partners in recent years. The INSPIRE framework has been identified as the basis for the intervention‐outcome framework for the present EGM (WHO‐INSPIRE, 2016).^1^ The INSPIRE framework supports the SDGs aiming to reduce significantly all forms of violence and related death rates everywhere. The seven INSPIRE strategies are supported by and contribute to activities aimed at achieving several SDG goals that target risk factors for violence against children, including those that address poverty, health, gender equality, education, safe environments and justice, and are therefore important to include in programming to prevent violence against all children (WHO‐INSPIRE, 2016).

The INSPIRE technical package presents strategies based on the best available evidence to help countries and communities intensify their focus on the prevention programmes and services with the greatest potential to reduce violence against children, applicable to their contexts. The ambition of INSPIRE is to provide a unifying approach to the field of violence prevention.^2^ The use of the INSPIRE framework to guide the design of the EGM will help purposively update the field of knowledge supporting INSPIRE and point to specific gaps and priorities that will need attention.

**Figure 2:**
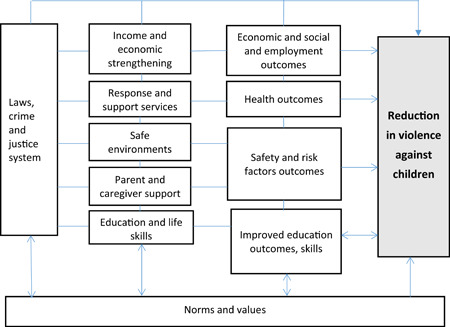
Conceptual framework for intervention and outcomes to reduce violence among children (Source: Authors' own design) [Color figure can be viewed at wileyonlinelibrary.com]

Figure 2 shows how this EGM builds on the seven strategies of the INSPIRE framework to develop intervention and outcome categories.

### 1.4 Scope of the EGM

The scope of this EGM is to capture all the effectiveness studies on reducing interpersonal violence and collective violence against children in LMICs. In so doing, the EGM will contribute to broadening the included interventions and outcomes in the INSPIRE framework to better reflect the state of evidence on violence against children, and independently provide an updated overview of knowledge and evidence gaps for the field.

The intended users are policymakers and funding organisations which are working to reduce interpersonal violence against children. The present EGM will provide an overview of all available evidence on the key outcome domains and interventions aimed at reducing violence against children in LMICs using an intervention‐outcome framework. It will identify areas in which there are existing bodies of synthesised knowledge to inform policy, and those areas in which there is little or no evidence synthesis.

The EGM will include studies of effectiveness of violence prevention strategies such as experimental and nonexperimental evaluations and systematic reviews. It does not aim to synthesise key messages from the available evidence but will only map the availability of quality evidence on childhood violence prevention based on the INSPIRE framework. As the EGM is focused on documenting availability of evidence, coverage of the breadth of strategies in the INSPIRE framework will be feasible and also strategic as identified above.

The scope of the EGM is defined by a framework of intervention and outcomes presented in two dimensions: the rows lists seven interventions and its subcategories, and seven outcome domains as columns. Each cell shows studies which contain evidence on that combination of intervention and outcome. Included studies are coded for additional characteristics as filters, such as population subgroup, context, country, region and type of violence, nature of violence and conflict‐affected regions.

### 1.5 Existing EGMs and/or relevant systematic reviews

The child welfare mega map (UNICEF Research Brief, 2018) was a basis for developing the present EGM. The mega map identified gaps in the area of violence against children in LMICs. The present EGM is an outcome of the findings of mega map which found, the evidence for protecting children from violence and exploitation is low in LMICs, nonetheless it is a priority area for policy and practice (UNICEF Research Brief, 2018).

There are four additional related ongoing maps. There is a map on child maltreatment and neglect which is restricted to HICs (Kornør, John, Brynhildur, Biedilæ, & Albers, 2017). A second map for LMICs focuses on child neglect (Sinha, Radhika, Jha, & John, 2018), whereas we will focus on child violence (we elaborate on this distinction below). The third map registered by Albers et al. (2019) is about institutional responses to child maltreatment, not focussing on any specific region. The fourth evidence and gap map is an unpublished work undertaken by Institute for Security Studies in partnership with the University of Johannesburg and Wits University to address violence against women and children in South Africa. There is no existing EGM for violence against children in LMICs on a global scale.

## 2 OBJECTIVES

The objective of this EGM is to provide an overview of the existing evidence base and gaps in evidence aimed at reducing violence against children in LMICs using an intervention‐outcome framework.

Consistent with this, the EGM will

I. Identify existing gaps in evidence to better inform future investment in research.
II. Identify clusters of primary studies that offer opportunities for evidence synthesis.
III. Identify, appraise and summarise existing evidence from systematic reviews of the effect of intervention to reduce violence against children.

## 3 METHODOLOGY

### 3.1 Defining evidence and gap maps

EGMs are evidence collections that map out existing and ongoing systematic reviews or primary studies in a sector or subsector. The EGMs are presented in two dimensions the rows list interventions and the column list outcome domains. Each cell shows studies which contain evidence on that combination of intervention and outcomes.

### 3.2 EGM framework

The EGM framework will inform the inclusion and exclusion criteria of the EGM. Here, we describe the population, intervention, comparison, outcomes (indicators) and study designs for the map (Table 1).

**Table 1:** Intervention categories, subcategories and examples

Intervention category	Subcategory	Examples
1. Laws, crime and justice	Law	Laws banning or increasing legal consequences for perpetration of violent (corporal) punishment or domestic violence, Laws criminalizing or increasing legal consequences for perpetration of sexual abuse and exploitation of children, Laws preventing or reducing substance misuse (advertisement, prices and coupons), Laws limiting youth access to firearms and other weapons and engagement in conflict, Family law/child protection legislations, Law on violence against children, Law on media content regulation
	Crime and justice system	Treatment programmes and other safeguards for juvenile offenders in the criminal justice system/gangs, strengthening police and judicial systems for child protection, Increasing access to informal justice, including community‐based legal aid and paralegal programmes, Adolescent intimate partner violence, Dating violence prevention
2. Norms and values	Community mobilisation programmes	Community‐wide interventions to raise awareness of child violence
	Bystander interventions	Interventions to empower bystanders to intervene and prevent violence
	Media campaigns including mass media and education	Media campaigns highlighting the issue of child violence
3. Safe environments	Making existing environments safe	Reducing violence by addressing “hotspots”, Interrupting the spread of violence, Improving the built environment (safe homes, schools), urban upgrading, Zoning strategies to reduce violence, Child protective services including safe orphanages/homes for children without guardianship
	Creating safe places	School WASH and infrastructure
4. Parent, child and caregiver support	Parent‐training and education—interventions that promote positive parenting practices	Parent and child support groups, Government agencies that coordinate/streamline all activities related to parenting and parent support, home visiting programmes, group parenting programmes, integrated parenting programmes
	Maternal/paternal mental health	Counselling and therapy for mental health support
	Peer/relationship training	Peer training, peer educators
5. Income and economic strengthening	Broad‐based social protection (economic transfers)	Conditional cash transfers, Unconditional cash transfers, Public works or cash‐for‐work, In‐kind transfers (food, vouchers and assets), Subsidies (housing, education and utility) or tax incentives, School feeding/free school lunch
	Income generating or savings/credit interventions	Group saving and loans (with and without additional components, e.g., gender equity training), Microfinance or credit (with and without additional components, e.g., gender norms training), Financial inclusion programmes (including savings programmes, financial literacy and access to banking), Livelihood or agricultural productivity programmes (including graduation programmes), Skills training/vocational or entrepreneurship programmes
	Insurance and welfare schemes	Health and other insurance, Employer and labour force benefits (including unemployment befits, maternity leave policies), Pensions and retirement benefits, Disability benefits
6. Response and support services	Counselling and therapeutic approaches	Specialised counselling and therapeutic services for victims of violence
	Screening and training	Reporting combined with interventions: Training the health professional/social workers/teachers to identify possible exposure or risk of exposure to violence
	Children in care	Includes alternative family care (foster or kinship care) or institutional care (orphanages, group homes, juvenile detention centres or residential treatment centres) interventions involving social welfare services, Shelters and crisis centres
	Media and communication	Awareness on access to services/reporting
7. Education and life skills	Gender transformative approaches	Including sexual and reproductive health education
	Life and social skills training	Violence prevention, Bullying prevention programmes, self‐defence, adolescent intimate partner violence/dating violence (interventions to prevent abusive behaviour in adolescent peer relationships)

#### 3.2.1 Population

The primary population of interest for this map is children and adolescents from LMICs. LMICs are defined by World Bank as low‐income economies—those with a Gross National Income (GNI) <$995; lower middle‐income economies—those with a GNI per capita between $996 and $3,895; and upper middle‐income economies—those with a GNI per capita between $3,896 and $12,055 (The World Bank, 2018). As defined in the INSPIRE handbook, “Child” means any person aged under 18 years (WHO‐INSPIRE, 2018). Following the guideline, children irrespective of their sex in the age group of ≤18 years will be included in the EGM. The age group is classified based on the WHO age criteria stated as follows: infanthood (<3 years of age), childhood (3–10 years), adolescence (10–18 years) (WHO‐GAA, 2017).

Population subgroup of interest includes girls, orphans and vulnerable children, children with disabilities, children belonging to ethnic minorities, child sex workers, child brides, isolated children/street children, children with HIV/acquired immunodeficiency syndrome (AIDS), migrants and children affected by conflict and humanitarian crises, including refugees and child soldiers.

#### 3.2.2 Intervention

This EGM is focused on effectiveness studies of interventions where the primary aim is the reduction of violence against children. The intervention‐outcome framework is based on the INSPIRE framework which identifies seven evidence‐based strategies to prevent violence against children viz. implementation and enforcement of laws; norms and values; safe environments; parent and caregiver support; income and economic strengthening; response and support services; and education and life skills (WHO‐INSPIRE, 2016). The intervention categories are based on the seven strategies of INSPIRE, however, the subcategories are based on pilot coding of key 30 studies, expert advice and discussion within the team and advisory members The deviation from the INSPIRE strategies for the first category viz. implementation and enforcement of laws, which was kept as laws, crime and justice in the present map. This deviation was based on the pilot coding of studies. For an EGM it is best practice to have fewer subcategories so that complex interventions can be coded correctly and avoid unnecessary overlap between subcategories.

Table 1 lists the intervention categories and subcategories. Examples of programme names are given in brackets. These are listed to aid with search and coding. They will not appear in the subcategory label in the map. The included interventions cover all main strategies to reduce violence against children outcomes operating across different contexts and relations where risks exist—from the individual level to families, communities, institutions and societies. The subcategories of interventions are largely based on INSPIRE guidelines, definitions for which are given as Appendix 1.

The intervention categories included in our map are:

1. Law, crime and justice system
2. Norms and values
3. Safe environment
4. Parent, child and caregiver support
5. Income and economic strengthening
6. Response and support services
7. Education and life skills.

### 3.3 Outcomes

The outcomes domains and subdomains will be the main categories and subcategories across the interventions in our map. The included studies should have a component of ending or reducing violence against children as a primary outcome. The seven main outcome categories are listed in the Table 2 given below:

**Table 2:** Outcome domains and subdomains

Outcome domains	Subdomains
1. Violence	Sexual
	Physical
	Emotional/psychological (financial abuse)
2. Norms, values, beliefs and attitudes	Belief on parenting practices
	Gender roles, attitudes and social norms
	Delinquent, violent and other risk‐taking behaviour (including reoffending, recidivism rates)
	Empowerment
3. Health	Substance abuse
	Child development and child mental health
	Maternal mental health
	Morbidity and mortality
	Sexual and reproductive health
4. Safety and risk factors	Social isolation (homeless and street connected children)
	Female genital mutilation (FGM) and child marriage
	Child labour/trafficking
	Safe environment/spaces
5. Economic and social	Poverty and food security
	Employment and labour force participation
	Savings and credit
	Social inclusion and gender equity
	Social discrimination
6. Cost analysis	Cost‐effectiveness
	Cost‐benefit
7. Education	School enrolment and attendance
	School performance
	WASH and infrastructure
	Gender roles and life skills

### 3.4 Criteria for including and excluding studies

#### 3.4.1 Types of study designs

Systematic reviews and impact evaluation or studies of effectiveness will be included for the EGM. Systematic reviews collating data on the effectiveness of interventions to prevent violence against children, child marriages and child labour will be included in the EGM. Systematic reviews containing one or more studies from LMICs will be included. Among primary studies, the best evidence is provided by randomised controlled trials (RCT), but for the present map, non‐RCTs will be used as a supplement to the available evidence in a particular area. Impact evaluation studies using quantitative data to evaluate the effectiveness of the intervention will be included based on the primary outcome of interest in the study viz. violence against children which includes direct violence inflicted on children, child marriages and child labour.

The dearth of RCTs is anticipated because in some cases or when addressing some issues related to violence, RCTs could be unethical. For non‐RCTs, a pretest of the outcome measure and relevant demographic characteristics, and/or statistical control of such characteristics will be present as a criteria for inclusion of pre–post test studies with or without comparison group. The study designs which will be included in the EGM are as follows: systematic reviews and impact evaluations done as—RCTs, quasi‐experimental studies, analytical observational (cohort studies), Modelling with empirically grounded parameters (instrumental variables), controlled before‐after studies and interrupted time‐series. Relevant on‐going studies will be included.

#### 3.4.2 Treatment of qualitative research

We do not plan to include qualitative research in the EGM.

#### 3.4.3 Types of settings

All types of settings, where interventions for violence against children were implemented, will be included in the EGM. On the basis of the framework, the settings may be: school, home, centre or facility, community and so on.

#### 3.4.4 Status of studies

Relevant ongoing studies will be included in the EGM.

The detailed eligibility criteria is given in Appendix 3.

### 3.5 Search strategy and status of studies

The search for EGM will be conducted in three stages:

**Table:** 

Stage	Step	Timeline
Stage 1	Pilot for screening and coding was done from the included studies in the INSPIRE seven strategies for Ending Violence Against Children (Know Violence in Childhood, 2017)	December 2018
Stage 2	This stage will include search of relevant systematic reviews and primary studies from academic databases and international organizations.	February 2019
Stage 3	This stage will include search on additional websites for grey literature after expert consultation.	April 2019

The search will be as comprehensive as possible, using (but not limited to) relevant bibliographic databases, web‐based search engines, websites of specialist organisations, bibliographies of relevant reviews, and targeted calls for evidence using professional networks or public calls for submission of articles.

Additionally, citation searches of included studies in Google Scholar, Scopus and Web of science will be performed. Reference lists of the included reviews will be reviewed (Appendix 2).

### 3.6 Databases

1. Systematic review databases
  – 3ie Systematic Review Database
  – Campbell Collaboration
  – Cochrane
  – Collaboration for Environmental Evidence
  – EPPI Centre Evaluation Database of Education Research
  – PROSPERO
  – Research for Development
  – Swedish Agency FOR Health Technology Assessment and Assessment of Social Services
  – Epistomonikos
2. Academic databases
  – Applied Social Sciences Index and Abstracts (ASSIA)
  – CABI's Global Health
  – Caribbean Child Development Centre Online Publication Database
  – CINAHL
  – EBSCO
  – EBSCOhost (Caribbean Search)
  – Econlit
  – Eldis
  – Embase
  – Emerald insight
  – ERIC
  – Google Scholar
  – International Bibliography of Social Sciences (IBSS)
  – IDEAS‐Repec
  – Popline
  – JGATE
  – JOLIS
  – JSTOR
  – MEDLINE
  – PsycINFO
  – PubMed
  – RedALyC (La Red de Revistas Científicas de América Latina, el Caribe, España y Portugal)
  – SafetyLit
  – SciELO
  – SCOPUS
  – Social Science Research Network (SSRN)
  – Sociological abstracts (ProQuest)
  – The National Bureau of Economic Research (NBER)
  – Web of Science
  – WHO's Global Health Library
3. International Organisations (Bilateral and multilateral)
  – DFID (including Research for Development (R4D)
  – ILO
  – IOM
  – Save the Children
  – UN Women
  – UNDP
  – UNFPA Evaluation Database
  – UNHCR
  – UNICEF
  – UNICRI
  – UNODOC
  – USAID
  – WHO/PAHO
4. Grey Literature search/websites
  – Abdul Latif Jameel Poverty Action Lab (J‐PAL)
  – Action against Hunger
  – Action Aid http
  – ADOLEC
  – African Development Bank
  – Africa‐Wide
  – Africaportal.org
  – African journals online (AJOLS)
  – Anulib
  – Association for the Development of Africa
  – British Library for Development Studies
  – CAF Development Bank of Latin America
  – CARE
  – CEPAL/ECLAC—Economic Commission for Latin America and the Caribbean
  – Child and Youth Finance International
  – CIFF
  – Clinton Foundation
  – Concern Worldwide Division for Social Policy & Development
  – Child Fund International
  – Fórum Brasileiro de Segurança Pública
  – CPC Learning Network
  – EU CORDIS
  – Gates Foundation
  – GreyNet International
  – Innovations for Poverty Action (IPA) Database
  – International Center for Research on Women (ICRW)
  – Inter‐American Development Bank (IADB)
  – International Food Policy Research Institute (IFPRI)
  – International Rescue Committee (IRC)
  – International Red Cross
  – IPC‐IG (Working papers)
  – Joanna Briggs Institute Evidence‐Based Practice Database
  – LLC/Centre for Human Services
  – LTSHM
  – MedCarib
  – Medecins Sans Frontières
  – Oak Foundation
  – One International
  – Opengrey
  – Organization of American States (OAS)
  – Overseas Development Institute
  – Project Concern
  – Proquest Dissertations & Theses
  – RAND Corporation
  – Sexual Violence Research Initiative (SVRI)
  – Social Care Online
  – UN Economic and Social Council UNESCO
  – UNICEF Innocenti Research Centre
  – University Research Co.
  – United Nations Population Fund
  – Urban Youth Evidence Synthesis
  – Valid International
  – Working Group on Early Childhood Development
  – World Bank Group (WBG)
  – Within WBG: Spanish Impact Evaluation Fund (SIEF)
  – Within WBG: Korean Trust Fund (KTF)
  – World Food Programme
  – World for World Organization
  – World Vision.

### 3.7 Screening and selection of studies

All titles and abstracts, and then full text, will be double screened, with a third‐party arbitrator or in the event of disagreement. The screening tool is given as Appendix 4. Automation will not be used.

### 3.8 Data extraction, coding and management

Coding will be done independently by two coders, with a third‐party arbitrator or in the event of disagreement.

### 3.9 Quality appraisal

The quality of the included systematic reviews will be assessed using AMSTAR 2 (Shea et al., 2017) and done independently by two reviewers. We will quality rate the primary studies (individual studies) based on the quality assessment tool for individual studies as described below. The quality assessment of all systematic reviews will be done and it will be aimed to be completed for primary studies within the set deadlines.

Due to the extensive grey literature aimed to be searched, the authors anticipate low/medium quality studies, from LMICs, can be included in the EGM. Studies will not be excluded based on the quality assessment and a colour coding will show the studies with different quality ratings on the map.

The tool used to assess study quality is shown in Appendix 5. This tool includes six criteria that are appropriate for the assessment of quantitative impact evaluations. These are as follows:

1. *Study design (potential confounders taken into account)*: Impact evaluations need either a well‐designed control group (preferably based on random assignment) or an estimation technique which controls for confounding and the associated possibility of selection bias.
2. *Power calculations*: Small sample size can result in an under‐powered study with a high risk of not detecting an effect from the intervention when there actually is one. The combination of under‐powered studies and publication bias can put an upward bias on the assessment of the overall effect size from a body of literature. The problem of sample size is addressed by conducting power calculations before the study to determine the required sample size. We will not use this item in the overall assessment of the study. However coding mention of power calculations signals the importance of both conducting and reporting power calculations.
3. *Attrition or losses to follow up*: Can be a major source of bias in studies, especially if there is differential attrition between the treatment and comparison group (called the control group in the case of RCTs) so that the two may no longer be balanced in pre‐intervention characteristics. The US Institute of Education Sciences What Works Clearing House (WWC) has developed standards for acceptable levels of attrition, in aggregate and the differential, which are applied here.
4. *Clear definition of violence*: for a study to be useful the study population must be clear, which means that the type and degree of violence should be clearly defined, preferably with reference to a widely‐used international standard.
5. *Description of intervention*: If the intervention is not well described then the evidence may be misinterpreted to support an intervention not actually supported by study findings. For example, “case work” or “shelter” are very broad descriptions, so more detail of the intervention is required so as to know what is actually being evaluated. We rate as low if the description is just named with no description, medium if there is a short description, and high if there is a detailed description. We do not use this item in the overall assessment of the study.
6. *Definition of outcomes*: Outcomes should be clearly defined so that study findings can be properly interpreted. So far as possible, unless a subjection perception is required, that questions should rely on objective factors, and utilise data collection instruments which have been validated for the context in which they are being applied. We rate as high of there is clear definition of the outcome and how it is being measured, or reference to an existing tool. Medium rating is given is if there is a brief description, and low if the outcome is named but not adequately described.
7. *Baseline balance*: Baseline balance means that the treatment and comparison groups have the same average characteristics at baseline, not only for outcomes but also for other factors which may affect the impact of the programme such as a prior history of parental alcohol abuse. We rate low confidence on study findings if baseline balance is not reported for non‐RCTs or it is reported and there is a significant difference of 10% or >10%, medium confidence if imbalance is between 5% and 10%, and high if an RCT or if imbalance is 5% or <5%.
8. *Overall assessment*: The overall assessment uses a weakest link in the chain principle so that the overall assessment is the lowest of assessment given to any of the relevant items. As mentioned above, not all items are used in this assessment. So the overall assessment is the lowest of the assessments for items 1, 4, 6 and 7.

### 3.10 Ethical considerations

Many difficult ethical dilemmas may arise when gathering information among children and adolescents. Given the obvious sensitive nature of research into violence against children, it is indeed challenging to conduct studies assessing violence against children. Despite experience, education, and good intentions, skilled professionals often find themselves questioning how to proceed with their activities with young people. Every person has a right to privacy, hence preserving the confidentiality of personal information is one of the fundamental principles governing the collection of data about individuals. In line with the above stated challenges, an ethical coding tool for measuring ethical standards of primary intervention/effects study was developed (Council for International Organisations of Medical Sciences, 2009; Population Council, 2005; Save the Children, 2004; WHO‐Ethical and Safety Recommendations, 2007). The nine item tool has five critical and four noncritical items. The scoring is done as ‘Strong ethical standards’, ‘Moderate ethical standards’ and ‘weak ethical standards’. The complete tool with scoring is given in Appendix 6. No study will be disqualified based on adequacy of ethical standards. Findings of the ethical considerations will be presented in report.

## 4 ANALYSIS AND PRESENTATION

### 4.1 Unit of analyses

Each entry in the map will be a systematic review or a primary study of effectiveness. The final EGM will identify the number of studies covered by the map in each sector or subsector.

### 4.2 Presentation

In addition to the interventions and outcomes, the following filters will be coded:

1. Population subgroups: The age group is classified based on the WHO age criteria stated as follows: infanthood (<3 years of age), childhood (3–10 years) and adolescence (10–18 years).
2. Context: Very high prevalence settings • Pregnant women, orphans, juvenile offenders, children with disabilities, children belonging to ethnic minorities, child sex workers, child brides, isolated children/street children, children with HIV/AIDS, male, female, LGBTQ, father, mothers.
3. Region: East Asia & Pacific, Europe & Central Asia, Latin America & Caribbean, Middle East & North Africa, North America, South Asia, Western Central Africa, Eastern Central Africa
4. Country
5. Economies: Low‐income economies, Lower‐middle‐income economies, Upper‐middle‐income economies, High‐income economies
6. Type of violence: Interpersonal violence
  A. Family (intimate partner/community)
  B. Collective violence: Social/political/economic (riot, conflict)
7. Nature of violence:
  A. Physical
  B. Sexual
  C. Emotional/psychological
  D. Polyvictimization.
8. Conflict‐affected regions: This will be defined based on Department for International Development (DFID) list of conflict affected regions updated as per current year (2018/2019).

### 4.3 Planned analysis

The EGM report shall provide tabulations or graphs of the number of studies, with accompanying narrative description, by

- Intervention category and subcategory
- Outcome domain and subdomain
- Table of “aggregate map” of interventions and outcomes
- Region
- Year
- Study type
- Type of violence
- Population subgroups.

## 5 STAKEHOLDER ENGAGEMENT

An advisory group was formed at the inception stage of this EGM. Feedback from the group members was received and assimilated in the framework plan at the title registration stage. The stakeholders will be engaged at all stages of the EGM to review and comment on interventions, studies, outputs, map findings and provide advice on dissemination channels.

This map is commissioned by UNICEF Innocenti who will take the lead in introducing the map into relevant policy discussions. The advisory group members for this EGM includes experts from all the pillars of INSPIRE framework as social, economic, education, health and wellbeing and they have been involved in working towards Violence against children in their fields including use of INSPIRE. Through a joint effort by UNICEF Innocenti and the advisory group members we are positive that this work will contribute to greater alignment of global and national efforts and will form the basis for improving the evidence base on violence against children in low and middle income countries.

The advisory group members for the EGM are as follows:

1. Dr. Karen Devries, Associate Professor in Social Epidemiology, LSHTM
2. Professor Lorraine Sherr, Head of Health Psychology Unit, Institute of Global Health, UCL
3. Professor J. (Julia) Sloth‐Nielsen, Professor, Department of Public Law and Jurisprudence, University of the Western Cape and Professor of Children's Rights in the Developing World, University of Leiden
4. Shivit Bakrania, Knowledge Management Specialist, Research Facilitation and Knowledge Management unit, UNICEF Innocenti Centre
5. Professor A.K. Shiva Kumar, Global Co‐Chair, Know Violence in Childhood
6. Professor Andrés Villaveces, Senior Scientist, Division of Violence Prevention, NCIPC, US Centers for Disease Control and Prevention, and Department of Epidemiology, University of North Carolina (UNC), Chapel Hill
7. Dr. Amber Peterman, Consultant, UNICEF Innocenti Centre and Associate Adjunct Professor at UNC Chapel Hill
8. Dr. Lucie Cluver, Professor of Child and Family Social Work, Centre for Evidence‐Based Social Intervention in the Department of Social Policy and Intervention, Oxford University
9. Professor Rebecka Lundgren, Deputy Director and Research Director, Institute for Reproductive Health, Georgetown University
10. Dr. Charlene Coore Desai, Resident Advisor, USAID Applying Science to Strengthen and Improve Systems (ASSIST) Project, Jamaica
11. Dr. Kerry Albright, Chief, Research facilitation and Knowledge management, UNICEF Innocenti Centre

## ROLES AND RESPONSIBILITIES

- Content:

Ramya Subrahmanian has an extensive experience in research, policy advocacy, training and teaching. She has experience in use of evidence across all of UNICEF's policy areas. In her previous capacity as Executive Director, Know Violence in Childhood, she oversaw the commissioning of over 45 new papers on violence prevention including systematic reviews on LMICs, as well as the publication of an updated annotated bibliography.

EGM methods:

Ashrita Saran and Howard White have previous experience in systematic review methodology, including searching, data collection, and theory‐based synthesis, which means they are proficient in carrying out the various processes in an EGM, such as search, eligibility screening, quality assessment and coding. They have undertaken an overview of approaches to mapping in a range of organisations. Jill Adona is an experienced screener and coder who has previously worked on Campbell Collaboration research projects. Jill has attended training workshops on evidence synthesis provided by both 3ie and Campbell. Prachi Pundir has experience in systematic reviews and has previously worked on systematic reviews and meta‐analysis with Public Health Evidence South Asia, Manipal and all authors are proficient in carrying out the various processes in an EGM, such as eligibility screening, quality assessment and coding.

Information retrieval:

Ashrita Saran, and Prachi Pundir have training in designing and implementing search strategies.

## SOURCES OF SUPPORT

The funding for this research is supported by UNICEF Office of Research ‐ Innocenti. The deliverable deadline for the EGM is August 31, 2019.

## DECLARATIONS OF INTEREST

The authors declare there is no conflict of interest.

## PRELIMINARY TIMEFRAME

Approximate date for submission of the EGM: July 31, 2019.

## PLANS FOR UPDATING THE EGM

Once completed, the EGM will be updated yearly, provided we have availability of funds. The lead author and/or the corresponding authors will be responsible for updating the EGM.

## Footnotes

1 In 2018, follow‐up resources on INSPIRE technical package were published. “INSPIRE indicator guidance and results framework” (UNICEF‐INSPIRE, 2018) and “INSPIRE handbook: action for implementing the seven strategies” were the two resources to improve the implementation and use of the guidelines (UNICEF, 2018; WHO, 2018). The “INSPIRE handbook” explains in detail how to choose and contextually implement interventions and “Indicator Guidance and Results Framework” is designed to help governments and agencies to monitor progress and track change over time when INSPIRE strategies are implemented.

2 Violence is rooted in social, economic and cultural factors that impact communities, families, relationships and the children's experiences of their daily lives. The social ecological model (given on page 17 of INSPIRE guidelines document; WHO‐INSPIRE, 2016) depicts this interplay of individual, relationship, community and societal factors. *Individual‐level factors* include biological and personal history aspects such as sex, age, education, income, habits; *Close‐relationship level factors* include parenting practices, family, separation, peers; *Community‐level factors* include schools, workplaces and neighbourhoods and *society‐level risk factors* include legal and social norms including health, economic, educational and social policies.

## APPENDIX 1 DEFINITIONS

Violence: Violence is understood to mean “all forms of physical or mental violence, injury or abuse, neglect or negligent treatment, maltreatment or exploitation, including sexual abuse”. It includes: maltreatment, bullying, youth violence, intimate partner violence, sexual violence and emotional or psychological violence.

- Maltreatment (including violent punishment) involves physical, sexual and psychological/emotional violence; and neglect of infants, children and adolescents by parents, caregivers and other authority figures, most often in the home but also in settings such as schools and orphanages.
- Bullying (including cyber‐bullying) is unwanted aggressive behaviour by another child or group of children who are neither siblings nor in a romantic relationship with the victim. It involves repeated physical, psychological or social harm, and often takes place in schools and other settings where children gather, and online.
- Youth violence is concentrated among children and young adults aged 10–29 years, occurs most often in community settings between acquaintances and strangers, includes bullying and physical assault with or without weapons (such as guns and knives), and may involve gang violence.
- Intimate partner violence (or domestic violence) involves physical, sexual and emotional violence by an intimate partner or ex‐partner. Although males can also be victims, intimate partner violence disproportionately affects females. It commonly occurs against girls within child marriages and early/forced marriages. Among romantically involved but unmarried adolescents it is sometimes called “dating violence”.
- Collective violence is defined as “the instrumental use of violence by people who identify themselves as members of a group—whether this group is transitory or has a more permanent identity—against another group or set of individuals, in order to achieve political, economic or social objectives.”

Various forms of collective violence have been recognised, including:

- Wars, terrorism and other violent political conflicts that occur within or between states.
- State‐perpetrated violence such as genocide, repression, disappearances, torture and other abuses of human rights.
- Organised violent crime such as banditry and gang warfare.
- Sexual violence includes nonconsensual completed or attempted sexual contact and acts of a sexual nature not involving contact (such as voyeurism or sexual harassment); acts of sexual trafficking committed against someone who is unable to consent or refuse; and online exploitation.
- Emotional or psychological violence includes restricting a child's movements, denigration, ridicule, threats and intimidation, discrimination, rejection and other nonphysical forms of hostile treatment.

Law, crime and justice system:

A) Laws: The system of rules which a particular country or community recognises as regulating the actions of its members and which it may enforce by the imposition of penalties.
B) Criminal justice system: The system of law enforcement that is directly involved in apprehending, prosecuting, defending, sentencing, and punishing those who are suspected or convicted of criminal offences (Criminal Justice System).

Norms and values: Aims to alter the social expectations that define “appropriate” behaviour for women and men, such as norms that dictate men have the right to control women, and which make women and girls vulnerable to physical, emotional and sexual violence by men (Norms and Values).

A) Community mobilisation programmes: Community mobilisation engages all sectors of the population in a community wide effort to address a health, social, or environmental issue. It brings together policy makers and opinion leaders, local, state, and federal governments, professional groups, religious groups, businesses, and individual community members. Community mobilisation empowers individuals and groups to take some kind of action to facilitate change.
B) Bystander interventions: Bystander Intervention is a social science model that predicts the likelihood of individuals (or groups) willing to actively address a situation they deem problematic. A bystander is anyone who observes any situation (Bystander Intervention).

Safe environments:

A) Child safe environments: Child safe environments are safe and friendly settings where children feel respected, valued and encouraged to reach their full potential (Child Safe Environment).
B) School WASH & Infrastructure: WASH is the collective term for Water, Sanitation and Hygiene. Due to their interdependent nature, these three core issues are grouped together to represent a growing sector. While each a separate field of work, each is dependent on the presence of the other. Buildings, classrooms, laboratories, and equipment are education infrastructure (Education Infrastructure).

Parent, child and caregiver support:

A) Parent‐training and education: Interventions that promote positive parenting practice
B) Parental mental health: Mental health is defined as a state of well‐being in which every individual realises his or her own potential, can cope with the normal stresses of life, can work productively and fruitfully, and is able to make a contribution to her or his community. Maternal and paternal refers to the affected child/children's mother or father respectively.
C) Peer/relationship training: Peer educators are typically the same age or slightly older than the group with whom they are working. Peer education is based on the reality that many people make changes not only based on what they know, but on the opinions and actions of their close, trusted peers. Peer educators can communicate and understand in a way that the best‐intentioned adults can't and can serve as role models for change.

Income and economic strengthening:

A) Broad‐based Social Protection (Economic transfers): Social protection systems help the poor and vulnerable cope with crises and shocks, find jobs, invest in the health and education of their children, and protect the aging population. It includes cash transfers, public works, in‐kind transfers, subsidies or school feeding.
  Cash transfers of two types: Conditional and unconditional cash transfers. Conditional cash transfer programmes give money to households on the condition that they comply with certain predefined requirements. These conditions can include, for example, up‐to‐date vaccinations, regular visits to a health care facility, regular school attendance by children, and complying with health and nutrition promotion activities (e.g., attending education sessions, taking nutritional supplements, etc.). Conditional cash transfer programmes are aimed at reducing poverty as well as breaking the cycle of poverty for the next generation through the development of human capital. Unconditional cash transfers include universal basic income interventions, where every citizen receives an unconditional basic income (Pega et al., 2017).
B) Income generating or savings/credit interventions: Income generation interventions attempt to address poverty, unemployment, and lack of economic opportunities to increase participants' ability to generate income and secure livelihoods (USAID).
C) Insurance and welfare schemes: Social insurance is comprised of programmes that minimise the negative impact of economic shocks on individuals and families. They include publicly provided or mandated insurance schemes against old age, disability, death of the main household provider, maternity leave and sickness cash benefits, and social‐health insurance. Social insurance programmes are contributory and beneficiaries receive benefits or services in recognition of contributions to an insurance scheme (The World Bank).

Response and support services:

A) Counselling and therapeutic approaches: Counselling is a learning‐oriented process, which occurs usually in an interactive relationship, with the aim of helping a person learn more about the self, and to use such understanding to enable the person to become an effective member of society.
B) Screening and training: Screening and reporting combined with interventions. Training the health professional/social workers/teachers for violence against children.
C) Creating safe spaces: Child friendly spaces can be defined as places designed and operated in a participatory manner, where children affected by natural disasters or armed conflict can be provided with a safe environment, where integrated programming including play, recreation, education, health, and psychosocial support can be delivered and/or information about services/supports provided.
D) Media and communication: Communication encompasses several areas including health journalism, entertainment, education, interpersonal communication, media advocacy, organisational communication, risk and crisis communication, social communication and social marketing (World Health Organization, 2019).

Education and life skills:

A) Sexual and reproductive health education: Good sexual and reproductive health is a state of complete physical, mental and social well‐being in all matters relating to the reproductive system. To maintain one's sexual and reproductive health, people need access to accurate information and the safe, effective, affordable and acceptable contraception method of their choice (UNFPA).
B) Life and social skills training: Are designed to help children and adolescents manage anger, resolve conflict and develop the necessary social skills to solve interpersonal problems without violence, and are usually implemented in school settings (Life and Social Skills Training).

## APPENDIX 2 SEARCH QUERY (MEDLINE)

We will prepare a search strategy using the title, abstract (textword) terms together with each database's indexing terminology, where available.

1. Developing Country Keywords:
  ((developing countr* or developing nation* or developing population* or “developing world” or less developed countr* or less developed nation* or less developed population* or “less developed world” or lesser developed countr* or lesser developed nation* or lesser developed population* or “lesser developed world” or under developed countr* or under developed nation* or under developed population* or “under developed world” or underdeveloped nation* or underdeveloped population* or “underdeveloped world” or middle income countr* or middle income nation* or middle income population* or low income countr* or low income nation* or low income population* or lower income countr* or lower income nation* or lower income population* or underserved countr* or underserved nation* or underserved population* or “underserved world” or under served countr* or under served nation* or under served population* or “under served world” or deprived countr* or deprived nation* or deprived population* or “deprived world” or poor countr* or poor nation* or poorer countr* or poorer nation* or poorer population* or developing economy* or less developed econom* or lesser developed econom* or under developed econom* or underdeveloped econom* or middle income econom* or low income econom* or lower income econom* or “low gdp” or “low gnp” or “low gross domestic” or “lower gdp” or “lower gnp” or “lower gross domestic” or “lower gross national” or lmic or lmics or “third world” or lami countr* or transitional countr* or L&MIC or LAMIC or LDC).mp. or LIC.ti,ab,kw.
  (Africa OR Algeria OR Angola OR Benin OR Botswana OR Burkina Faso OR Burundi OR Cameroon OR “Cape Verde” OR “Central African Republic” OR Chad OR “Democratic Republic of the Congo” OR “Republic of the Congo” OR Congo OR “Cote d'Ivoire” OR “Ivory Coast” OR Djibouti OR Egypt OR “Equatorial Guinea” OR Eritrea OR Ethiopia OR Gabon OR Gambia OR Ghana OR Guinea OR Guinea‐Bissau OR Kenya OR Lesotho OR Liberia OR Libya OR Madagascar OR Malawi OR Mali OR Mauritania OR Morocco OR Mozambique OR Namibia OR Niger OR Nigeria OR Rwanda OR “Sao Tome” OR Principe OR Senegal OR “Sierra Leone” OR Somalia OR Somaliland OR “South Africa” OR “South Sudan” OR Sudan OR Swaziland OR Tanzania OR Togo OR Tunisia OR Uganda OR Zambia OR Zimbabwe).ti,ab,kw.
  (“South America” OR “Latin America” OR “Central America” OR Mexico OR Argentina OR Bolivia OR Brazil OR Chile OR Colombia OR Ecuador OR Guyana OR Paraguay OR Peru OR Suriname OR Uruguay OR Venezuela OR Belize OR “Costa Rica” OR “El Salvador” OR Guatemala OR Honduras OR Nicaragua OR Panama).ti,ab,kw.
  (“Middle East” OR “South‐East Asia” OR “Indian Ocean Island*” OR “South Asia” OR “Central Asia” OR Caucasus OR Afghanistan OR Azerbaijan OR Bangladesh OR Bhutan OR Burma OR Cambodia OR China OR Georgia OR India OR Iran OR Iraq OR Jordan OR Kazakhstan OR Korea OR “Kyrgyz Republic” OR Kyrgyzstan OR Lao OR Laos OR Lebanon OR Macao OR Mongolia OR Myanmar OR Nepal OR Oman OR Pakistan OR Russia OR “Russian Federation” OR “Saudi Arabia” OR Bahrain OR Indonesia OR Malaysia OR Philippines OR Sri Lanka OR Syria OR “Syrian Arab Republic” OR Tajikistan OR Thailand OR Timor‐Leste OR Timor OR Turkey OR Turkmenistan OR Uzbekistan OR Vietnam OR “West Bank” OR Gaza OR Yemen OR Comoros OR Maldives OR Mauritius OR Seychelles).ti,ab,kw.
  (“Pacific Islands” OR “American Samoa” OR Fiji OR Guam OR Kiribati OR “Marshall Islands” OR Micronesia OR New Caledonia OR “Northern Mariana Islands” OR Palau OR “Papua New Guinea” OR Samoa OR “Solomon Islands” OR Tonga OR Tuvalu OR Vanuatu).ti,ab,kw
  (“Eastern Europe” OR Balkans OR Albania OR Armenia OR Belarus OR Bosnia OR Herzegovina OR Bulgaria OR Croatia OR Cyprus OR “Czech Republic” OR Estonia OR OR Kosovo OR Latvia OR Lithuania OR Macedonia OR Malta OR Moldova OR Montenegro OR OR Romania OR Serbia OR “Slovak Republic” OR Slovakia OR Slovenia OR Ukraine).ti,ab,kw.
  (Afghanistan OR Albania OR Algeria OR American Samoa OR Angola OR Armenia OR Azerbaijan OR Bangladesh OR Belarus OR Belize OR Benin OR Bhutan OR Bolivia OR Bosnia and Herzegovina OR Botswana OR Brazil OR Bulgaria OR Burkina Faso OR Burundi OR Cabo Verde OR Cambodia OR Cameroon OR Central African Republic OR Chad OR China OR Colombia OR Comoros OR Congo, Dem. Rep. OR Congo, Rep. OR Costa Rica OR Côte d'Ivoire OR Cuba OR Djibouti OR Dominica OR Dominican Republic OR Ecuador OR Egypt, Arab Rep. OR El Salvador OR Equatorial Guinea OR Eritrea OR Ethiopia OR Fiji OR Gabon OR Gambia, The OR Georgia OR Ghana OR Grenada OR Guatemala OR Guinea OR Guinea‐Bissau OR Guyana OR Haiti OR Honduras OR India OR Indonesia OR Iran, Islamic Rep. OR Iraq OR Jamaica OR Jordan OR Kazakhstan OR Kenya OR Kiribati OR Korea, Dem. People's Rep. OR Kosovo OR Kyrgyz Republic OR Lao PDR OR Lebanon OR Lesotho OR Liberia OR Libya OR Macedonia, FYR OR Madagascar OR Malawi OR Malaysia OR Maldives OR Mali OR Marshall Islands OR Mauritania OR Mauritius OR Mexico OR Micronesia, Fed. Sts. OR Moldova OR Mongolia OR Montenegro OR Morocco OR Mozambique OR Myanmar OR Namibia OR Nauru OR Nepal OR Nicaragua OR Niger OR Nigeria OR Pakistan OR Papua New Guinea OR Paraguay OR Peru OR Philippines OR Romania OR Russian Federation OR Rwanda OR Samoa OR São Tomé and Principe OR Senegal OR Serbia OR Sierra Leone OR Solomon Islands OR Somalia OR South Africa OR South Sudan OR Sri Lanka OR St. Lucia OR St. Vincent and the Grenadines OR Sudan OR Suriname OR Swaziland OR Syrian Arab Republic OR Tajikistan OR Tanzania OR Thailand OR Timor‐Leste OR Togo OR Tonga OR Tunisia OR Turkey OR Turkmenistan OR Tuvalu OR Uganda OR Ukraine OR Uzbekistan OR Vanuatu OR Venezuela, RB OR Vietnam OR West Bank and Gaza OR Yemen, Rep. OR Zambia OR Zimbabwe).mp.
2. Population Keywords:
  (child* OR young child* OR pre‐schooler* OR kindergarten* OR early child or childhood or early year*).ti,ab,kw.
  (juvenile* OR minors or youth OR “young adult*” OR “young wom$n” OR “young m$n” OR girl* OR boy* OR (school adj6 student*) OR teen* OR schoolgirl* OR schoolboy*).ti,ab,kw
  (pupil* OR student* OR partner* OR spouse* OR peer* OR boyfriend* OR boy friend* OR girlfriend* or girl friend* OR acquaintance* OR non stranger* OR nonstranger*).ti,ab,kw.
  (adolescen* OR boy$1 OR boyhood OR girl* OR teen* OR preteen* OR pubescen* OR prepubescen* or youth* OR juvenile* OR preteen* OR pre teen* OR young people* OR young person* OR early adult* OR young adult* OR infan* OR baby or babies OR neonate* OR newborn*).ti,ab,kw.
3. Violence Keywords:
  (Violen* or Aggress*) NOT (“cancer* OR disease* OR neoplasm*”).ti,ab,kw.
  (abus* OR maltreat* OR mistreat* OR ill‐treat* OR harm* OR victimi* OR ill‐use* OR misuse* ORmis‐use* OR oppress* OR violat* OR batter* OR bully OR bullied).ti,ab,kw.
  (Sexual* adj5 (assault or harassment or exploitation or traffic* or slave*)).ti,ab,kw.
  (human traffic* OR harmful traditional practice* OR female genital mutilation OR FGM OR female genital cutting OR slavery OR forced prostitution OR forced marriage* OR early marriage*).ti,ab,kw.
  (harsh parent* OR corporal punishment OR beat* OR physical discipline or paddling OR spank* OR bully* OR fight* OR fought OR assault*).ti,ab,kw.
4. Study design Keywords:
  (systematic* OR synthes*) adj3 (research OR evaluation* OR finding* OR thematic* OR report OR descriptive OR explanatory OR narrative OR meta* OR review* OR (map adj3 (evidence or gap)).ti,ab,kw.
  (“meta regression” OR “meta synth*” OR “meta‐synth*” OR “meta analy*” OR “metaanaly*” OR “meta‐analy*” OR “metanaly*” OR “metaregression” OR “metaregression” OR “methodologic* overview” OR “pool* analys*” OR “pool* data” OR “quantitative* overview” OR “research integration”).ti,ab,kw.
  (“impact evaluation” OR counterfactual OR experiment* OR “quasi‐experimental” OR “discontinuity design” OR “discontinuity regression” OR “regression discontinuity” OR “fixed effect*” OR regression OR “difference* in difference*” OR “double differenc*” OR “instrumental variable*” OR "propensity score matching" OR matching OR “propensity weight*” OR “interrupted time‐series” OR “panel data” OR “double robust” OR “random* control*” OR randomi?ation OR “random* trial*” OR “control group” OR “impact assessment” OR “econometric analys*” OR “cross‐sectional data” OR “difference‐in‐difference).ti,ab.kw.
  (cross‐sectional OR observational OR retrospective OR longitudinal OR survey* OR cohort OR follow up OR follow up OR case‐control OR case control OR mixed method OR mixed‐method).ti,ab.sh.

**Example of search in Medline:**

**Table:**

Results	Type	Actions
1	(Violen* or Aggress*).mp. not "cancer* OR disease* OR neoplasm* ".ti,ab,kw. [mp = title, abstract, full text, caption text]	389,984
2	(abus* or maltreat* or mistreat* or ill‐treat* or harm* or victimi* or ill‐use* or misuse* or mis‐use* or oppress* or violat* or batter* or bully or bullied).ti,ab,kw.	79,845
3	(Sexual* adj5 (assault or harassment or exploitation or traffic* or slave*)).ti,ab,kw.	1,762
4	(human traffic* or harmful traditional practice* or female genital mutilation or FGM or female genital cutting or slavery or forced prostitution or forced marriage* or early marriage*).ti,ab,kw.	645
5	(harsh parent* or corporal punishment or beat* or physical discipline or paddling or spank* or bully* or fight* or fought or assault*).ti,ab,kw.	21,755
6	((systematic* or synthes*) adj3 (research or evaluation* or finding* or thematic* or report or descriptive or explanatory or narrative or meta* or review*)).mp. or (map adj3 (evidence or gap)).ti,ab,kw. [mp = title, abstract, full text, caption text]	257,933
7	(“meta regression” or “meta synth*” or “meta‐synth*” or “meta analy*” or “metaanaly*” or “meta‐analy*” or “metanaly*” or “metaregression” or “metaregression” or “methodologic* overview” or “pool* analys*” or “pool* data” or “quantitative* overview” or “research integration”).ti,ab,kw.	53,728
8	(random$ or placebo$ or single blind$ or double blind$ or triple blind$).ti,ab.	292,426
9	(systematic$ adj2 (review$ or overview)).ti,ab.	54,334
10	(quantitativ$ adj5 synthesis$).tw.	8,719
11	(quantitativ$ adj5 review$).tw.	11,224
12	(cross‐sectional or observational or retrospective or longitudinal or survey* or cohort or follow up or follow up or case–control or case control or mixed method or mixed‐method).ti,ab,kw.	691,354
13	(developing countr* or developing nation* or developing population* or “developing world” or less developed countr* or less developed nation* or less developed population* or “less developed world” or lesser developed countr* or lesser developed nation* or lesser developed population* or “lesser developed world” or underdeveloped countr* or underdeveloped nation* or underdeveloped population* or “underdeveloped world” or underdeveloped nation* or underdeveloped population* or “underdeveloped world” or middle income countr* or middle‐income nation* or middle‐income population* or low‐income countr* or low‐income nation* or low‐income population* or lower income countr* or lower income nation* or lower income population* or underserved countr* or underserved nation* or underserved population* or “underserved world” or under served countr* or under served nation* or under served population* or “under served world” or deprived countr* or deprived nation* or deprived population* or “deprived world” or poor countr* or poor nation* or poorer countr* or poorer nation* or poorer population* or developing economy* or less developed econom* or lesser developed econom* or under developed econom* or underdeveloped econom* or middle income econom* or low income econom* or lower income econom* or “low gdp” or “low gnp” or “low gross domestic” or “lower gdp” or “lower gnp” or “lower gross domestic” or “lower gross national” or lmic or lmics or “third world” or lami countr* or transitional countr* or L&MIC or LAMIC or LDC).mp. or LIC.ti,ab,kw. [mp = title, abstract, full text, caption text]	185,155
14	(child* or young child* or pre‐schooler* or kindergarten* or early child or childhood or early year*).ti,ab,kw.	336,982
15	(juvenile* or minors or youth or “young adult*” or “young wom$n” or “young m$n” or girl* or boy* or (school adj6 student*) or teen* or schoolgirl* or schoolboy*).ti,ab,kw.	105,160
16	(pupil* or student* or partner* or spouse* or peer* or boyfriend* or boy friend* or girlfriend* or girl friend* or acquaintance* or non stranger*).mp. or nonstranger*.ti,ab,kw. [mp = title, abstract, full text, caption text]	1,124,674
17	(adolescen* or boy$1 or boyhood or girl* or teen* or preteen* or pubescen* or prepubescen* or youth* or juvenile* or preteen* or pre teen* or young people* or young person* or early adult* or young adult* or infan* or baby or babies or neonate* or newborn*).ti,ab,kw.	286,117
18	1 or 2 or 3 or 4 or 5	471,292
19	6 or 7 or 8 or 9 or 10 or 11 or 12	1,120,191
20	18 and 19	130,953
21	13 and 20	9,245
22	14 or 15 or 16 or 17	1,524,337
**23**	**21 and 22**	**5,776**

## APPENDIX 3 DETAILED ELIGIBILITY CRITERIA

**Table:**

	Include	Exclude
Literature type	Published journal articles	Commentary or conceptual papers
	Grey literature including technical reports which report quantitative data related to effectiveness of interventions for violence against children	Editorial
		Conference proceedings
	Technical reports/working papers	Case studies
Study design	All study designs must include quantitative data	Purely qualitative studies will be excluded regardless of design.
	Permissible study designs include:	
		Descriptive studies (cross‐sectional studies) reporting data narratively, but do not give statistical analysis
	Systematic reviews	
		Literature reviews
	Metanalysis/meta regressions	
	Randomised controlled trial	
	Modelling with empirically grounded parameters/econometric studies (Regression discontinuity, propensity score or other matching techniques, difference in difference)	
	Instrumental variables	
	Other matching designs	
	Rigorous quasi‐experimental design/quasi‐experimental	
	Natural experiments	
	Single‐subject design	
	Analytical observational	
	Before‐after studies	
	Time‐series	
Population	Children in the group of less than or equal to 18 years	Studies with target age group or beneficiaries (indirect) above 18 years of age.
	Age group is classified based on the WHO age criteria stated as follows: infanthood (<3 years of age), childhood (3–10 years), adolescence (10–18 years).	
	Children from LMICs as defined by World Bank classification.	
	We will include studies that have age‐group including both children and adults provided the interventions are directed towards children (having age range 0–18 years, but will exclude studies with beneficiary population above 18 years).	
	For systematic reviews with global focus, we will:	
	Exclude the systematic reviews that include studies only from high income even if they did not have any search restrictions.	
	Population subgroup of interest includes: orphans, children with disabilities, children belonging to ethnic minorities, child sex workers, child brides, isolated children/street children, children with HIV/AIDS and children in conflict and humanitarian settings.	
Interventions	We will include studies with interventions that aim reduce violence against children as a primary focus.	Interventions that focus on outcome related to child neglect, negligent behavior will be excluded
	These interventions will be based on INSPIRE guidelines and include categories as:	Interventions not focused on children
	1. Laws, crime and justice: Law, Crime and justice system.	
	2. Norms and values: Community mobilization programmes, bystander interventions, media campaigns including mass media and education.	
	3. Safe environments: Making existing environments safe (through design changes), creating safe places, hotspot evaluation approaches	
	4. Parent, child and caregiver support: Parent‐training and education—interventions that promote positive parenting practices, maternal/paternal mental health, peer/relationship training, parent and child support groups, government agencies that coordinate/streamline all activities related to parenting and parent support.	
	5. Income and economic strengthening: Broad‐based social protection (economic transfers), Income generating or savings/credit interventions, insurance and welfare schemes	
	6. Response and support services: Counselling and therapeutic approaches, screening and training, children in care, media and communication	
	7. Education and life skills: Gender transformative approaches, life and social skills training	

Outcomes	We will include studies that aim to reduce violence against children as a primary outcome and including following:	
	1. Violence: Sexual violence, physical violence, emotional/psychological violence (Financial abuse)	
	2. Norms, values, belief and attitude: Belief on parenting practices, gender roles, delinquent, violent and other risk‐taking behavior (including reoffending, recidivism rates), empowerment	
	3. Health: Substance abuse, child development and child mental health, maternal mental health, morbidity and mortality, sexual and reproductive health	
	4. Safety and risk factors: Social isolation (homeless and street connected children), female genital mutilation (fgm) and child marriage, child labour/trafficking, safe environment/spaces	
	5. Economic and social: Poverty and food security, employment and labour force participation, savings and credit, social discrimination (caste, race, ethnicity), social inclusion and gender equity	
	6. Cost Analysis: Cost‐effectiveness, cost‐benefit Education	
	7. School enrolment & attendance: School performance, WASH & Infrastructure, gender roles and life skills	

## APPENDIX 5 CRITICAL APPRAISAL TOOL

**Table:**

Item		Point in time (where applicable)	Rating
1a	Study design (Potential confounders taken into account)	End of intervention	High confidence: RCT, RDD, ITT, IV
			Medium confidence: DiD with matching, PSM
			Low confidence: other matching
1b	Study design (Potential confounders taken into account)	Longest follow up (if applicable)	Study design may change at post endline follow up, usually loss of RCT as control becomes treated. Same codes as 1a
2	Masking or blinding (RCTs only)		High confidence: any blinding or any mention of blinding
			Medium confidence: no blinding
			Low confidence is not used for this item
3	Power calculations are reported		High confidence: any mention of power calculations as basis for sample size
			Medium confidence: no mention of power calculations
			Low confidence is not used for this item
4a	Losses to follow up are presented and acceptable*	End of intervention	High: attrition within IES bounds
			Medium: attrition close to IES bounds
			Low: attrition not reported or attrition outside IES bounds
			N/A for ex post studies
4b	Losses to follow up are presented and acceptable*	Longest follow up (if applicable)	High: attrition within IES bounds
			Medium: attrition close to IES bounds
			Low: attrition not reported or attrition outside IES bounds
			N/A for ex post studies
5	Intervention if clearly defined		High confidence: intervention clearly and fully described
			Medium confidence: brief description of intervention
			Low confidence: intervention named but not described, or not named
6	Outcome measures are clearly defined and reliable		High confidence: outcome measure clearly and fully described, preferably with reference to validation
			Medium confidence: brief description of outcome
			Low confidence: outcome named but not described
7	Baseline balance (N.A. for before vs. after)		High confidence: RCT or baseline balance report and satisfactory (imbalance on 5% or <5%)
			Medium confidence: Imbalance between 5% and 10%
			Low confidence: Baseline balance not reported, or reported and lack of balance on 10% or >10%
	Overall confidence in study findings	End of intervention	Lowest rating across items 1a, 4a, 6 and 7
	Overall confidence in study findings	Longest follow up (if applicable)	Lowest rating across items 1b, 4b, 6 and 7 (N/A if 1b and 4b N/A)

*Maximum acceptable rate of differential attrition for each overall attrition rate (Source: Deke, Sama‐Miller & Hershey, 2015).

DiD, difference in difference; IES, Institute of Education Studies; ITT, intention to treat; IV, instrumental variable; PSM, Propensity Score Matching; RCT, randomised controlled trial; RDD, regression disconyinuity design.

## APPENDIX 6 ETHICS CODING TOOL

“Ethics” can be defined as a system or code of moral values that provides rules and standards of conduct. The three primary ethical principles that should guide all inquiries involving human beings (including methods used to collect information) are as follows:

1) Respect for persons, which relates to respecting the autonomy and self‐determination of participants, and protecting those who lack autonomy, including by providing security from harm or abuse.
2) Beneficence, a duty to safeguard the welfare of people/communities involved, which includes minimising risks and assuring that benefits outweigh risks.
3) Justice, a duty to distribute benefits and burdens fairly.

The nine item tool has five critical and four noncritical items (rating given below):

Ethical adequacy tool:

**Table:**

	Question	Response
1	Does the study have an ethical committee approval?*	Yes/No/Can't say
2	Does the study mention about training given to data collectors?*	Yes/No/Can't say
3	Does the study mention about informed consent from participants/providing information about study to participants?*	Yes/No/Can't say
4	Was the intervention given/considered to control arm after the study was complete/as a part of study design?	Yes/No/Can't say
5	Does the study declare researcher interest and funding?*	Yes/No/Can't say
6	Does the study mention how confidentiality/anonymity of participants was preserved?*	Yes/No/Can't say
7	Does the study mention about consideration given to respondents' willingness to disclose victimization?	Yes/No/Can't say
8	Does the study mention about dissemination policy (right to result, potential risks, purpose and benefit)?	Yes/No/Can't say
9	Does the study mention about engagement with local community/government agencies/research partners in conflict settings?	Yes/No/Can't say

*Critical item.

Rating ‘Strong ethical standards’ if response is yes for more than or equal to 3 critical items. Rating ‘Moderate ethical standards’ if response is yes for at least 2 critical items.Rating ‘Low ethical standards’ if critical item is marked as ‘yes’ for one or no critical item(s)
